# Application of Narrative Nursing Combined With Focused Solution Model to Anxiety and Depression in Patients With Lung Tumor During Perioperative Period

**DOI:** 10.3389/fsurg.2022.858506

**Published:** 2022-04-08

**Authors:** Jing Feng, Ling Ge, Fengxia Jin, Li Jiang

**Affiliations:** ^1^Department of Thoracic Surgery, Shanghai Chest Hospital, Shanghai Jiaotong University, Shanghai, China; ^2^Thoracic Surgical Intensive Care Unit, Shanghai Chest Hospital, Shanghai Jiaotong University, Shanghai, China

**Keywords:** focus resolution mode, narrative care, depression, anxiety, lung tumor

## Abstract

**Background:**

In the face of the dual pressure of disease and operation, patients with lung tumors in the perioperative period often have adverse psychological states such as anxiety and depression. There are many risk factors affecting the psychological state of patients in the perioperative period, and there is still a lack of effective nursing countermeasures in clinical practice.

**Materials and Methods:**

We accessed our institutional database and retrospectively selected all patients with lung tumors who underwent surgical treatment between August 2018 and December 2018. Multivariate Logistic regression model was used to analyze the risk factors affecting the psychological state of patients during the perioperative period, and the HAD score, medication behavior, INR monitoring behavior and life behavior before and after narrative nursing combined with focused solution model nursing were compared with those of patients receiving conventional nursing plan.

**Results:**

According to the inclusion and exclusion criteria, 148 cases of lung tumor patients undergoing surgical treatment were studied in this study. There were 45 cases without anxiety and depression and 103 cases with anxiety and depression in 148 patients. Income, medical environment, worry about work, family members' psychological state, family care, sleep quality, mental history, worry about postoperative pain, patients' knowledge of the diagnosis of the condition and the nature of the tumor were the single factors that affected the psychological state of patients with pulmonary tumor during perioperative period (*P* < 0.05). Multivariate analysis showed that income status, sleep quality, psychological status of family members, fear of postoperative pain and nature of tumor were the independent risk factors for psychological status of patients with lung tumor during perioperative period (*P* < 0.05). There was no difference in HAD score, medication behavior, INR monitoring behavior and life behavior score between the two groups when entering the group (*P* > 0.05). At the time of discharge, the HAD scores of the two groups were decreased, and those in the study group were lower than those in the control group (*P* < 0.05). At the time of discharge, medication behavior, INR monitoring behavior and life behavior of the two groups were increased, and the study group was higher than the control group (*P* < 0.05).

**Conclusion:**

Income status, sleep quality, fear of postoperative pain, Patient's knowledge of their condition and nature of tumor are the independent risk factors affecting the psychological state of patients with lung tumor during perioperative period. Narrative nursing combined with focused solution model can effectively improve the anxiety and depression status of patients with lung tumor during perioperative period and enhance their compliance behavior, which is worthy of promotion.

## Introduction

Lung tumors, as one of the most common tumors in clinic. It originate from respiratory epithelial cells such as bronchi, bronchioles and alveoli, and can be classified into benign and malignant tumors, with malignant being more common (1). There are two types of benign tumors and malignant tumors, and malignant tumors are more common in clinical practice. Surgical resection is the first choice for radical treatment of lung tumors. Studies have shown that most patients with lung cancer in the perioperative period mostly have adverse psychological states of anxiety and depression, and a few of them even have suicidal thoughts (2). However, the psychological state of patients is often closely related to the quality of life, the clinical efficacy of patients who adopt a negative psychological response to clinical treatment is often unsatisfactory. So it is important to explore the influencing factors of anxiety and depression in patients with lung cancer during perioperative period and formulate relevant nursing countermeasures (3).

Narrative nursing is a new nursing mode innovatively developed on the basis of nursing theory. It can discover nursing problems by narrating with patients, which is more humanized than the traditional nursing model (4). Solution focused approach (SFA) is a psychological intervention method with the purpose of mining patients' own resources and capabilities, which can enhance patients' confidence in treatment and improve the clinical outcomes (5). In the past, it has not been reported that the two care modes were applied to disease care at the same time. To further improve the level of care for patients with lung cancer during the perioperative period in our hospital, we combined the two care modes and observed the care anxiety of patients, which is reported below.

## Materials and Methods

This study has been approved by the Ethics Committee of our hospital. All patients have informed and consented, and all the data have been confirmed.

We accessed our institutional database and retrospectively screened the case data of lung tumors who underwent surgery between August 2018 and December 2018. Inclusion criteria were as follows: (i) Patients with lung tumors confirmed by clinical, imaging, endoscopic and pathological examination; (ii) Primary school education or above; (iii) Patients with clear consciousness and no communication disorders. Exclusion criteria: (iv) Patients with severe heart, liver, kidney and nervous system diseases; (v) Patients with a history of mental illness or family history; (vi) Patients with a history of malignant tumor treatment.

The medical records of all patients were retrieved, and the baseline characteristics of all patients were collected. Including the patient's age, gender, marital status, income status, medical environment, worries about work, psychological state of family members, family care level, sleep quality, history of mental illness, worries about postoperative pain, patient's knowledge of their condition and nature of tumor. Amog them, the Pittsburghsleepqualityindex (PSQI) was used to assess sleep quality.

Perioperative Anxiety and Depression status of all patients was assessed by the Hospital Anxiety and Depression Scale (HAD) (6). HAD was composed of two sub-tables, anxiety and depression, each of which contained seven items, and each item was scored according to the Liker four-level scoring system, with the scoring range of each scale ranging from 0 to 21 points. Patients with HAD score <8 points were considered as no-anxiety and depression, and patients with HAD score ≥ 8 points were considered as have anxiety and depression.

The investigator explained the purpose of the study to the surgical patients who had been included in the study. With the consent of the patients, the investigator himself/herself completed the questionnaire and general information questionnaire. Those who could not complete the questionnaire independently were filled in by the investigator through questions and answers.

The general information questionnaire was first filled out at the time of admission and the HAD scale was completed 1 day before surgery. All questionnaires were collected on the same day. According to the HAD evaluation results at the time of admission, the patients were divided into with anxiety and depression group and without anxiety and depression group.

All operations were performed according to the standard protocol. According to the differences of perioperative nursing methods, the patients were divided into a study group and a control group.

According to the doctor's advice, the nursing staff should guide the patient to train abdominal breathing, bed rest and defecation before operation. At the same time, the patients were given posture care, airway management, early rehabilitation exercise, diet care, wound care, drainage tube care and health education after operation.

On this basis, patients in the study group were given narrative care combined with SFA. (i) Setting up a nursing group: With the head nurse of the department as the team leader, a nursing group consisting of one resident, one psychological consultant, two supervisor nurses and several responsible nurses was established. (ii) The basic data of the patients were analyzed within 1-2 d after admission, and one-on-one communication was conducted based on the basic data of patients. In the process of communication with patients, nurses should follow the principle of narrative nursing. First, the nurses should help patients to treat the problems brought by the disease rationally, guide patients to explore how the problem is formed and find the reasons behind the problem. Second, the occurrence of tumor is bound to cause a serious decline in the quality of life of patients, when we can use the positive guidance method, and patients with hypothetical reasoning. Discuss hypothetical questions with the patient, for example: How was life before the tumor? What is the effect of surgical treatment on? What is the negative effect of anxiety and depression on anticoagulation therapy? How to improve compliance behavior and so on. In the process of answering these questions, we can guide patients how to resolve anxiety and depression, form the habit of complying with the doctor, and actively cooperate with the treatment. At the same time, nurses can help patients to find an external witness to improve their treatment compliance when necessary, so that they can better receive treatment by building a sense of ceremony. Third, treatment documents are organized. According to the real feelings of patients, language, text and audio are used to help patients achieve relevant phased goals, and patients are helped to reshape their identity and identify with themselves according to their biased values and life goals. (iii) Construction goal: According to the clinical characteristics and psychological status of patients undergoing pulmonary tumor surgery, nursing team members can work with patients to construct feasible overall intervention goals, specifically HAD <8 points, life behavior score ≥ 11 points, and total compliance behavior score ≥ 26 points. In addition, goals can be changed timely according to the specific behaviors of patients during implementation. (iv) The anxiety and depression degree and compliance behavior of patients were evaluated every 1 d, and compared with the scores before the start of nursing and the last test. The causes of changes in related indicators were analyzed, and the intervention plan was timely adjusted to guide the patients to make targeted improvements. The condition that the target value of patients reached the standard was evaluated weekly, and those who were satisfied with the evaluation results were given affirmation and praise. They constantly adjusted the potential of patients themselves and worked hard to achieve the ultimate intervention goal.

The HAD scale was used to assess the anxiety and depression states of the two groups. The compliance behavior of the two groups was assessed using the Compliance Behavior Scale, which included medication behavior, INR monitoring behavior and life behavior. Each item was scored using the Liker 4-level scoring system (7). The higher the score was, the better the compliance was. All observations were assessed at admission and discharge.

SPSS22.0 software was used for processing. The continuous variable data of experimental data were expressed as mean standard deviation ($\bar{x}$ ± s)and adopted *t*-test. The classified variable data and descriptive analysis were expressed as (%) and adopted χ^2^ test. Multivariate Logistic regression model was used to analyze the significant factors in single factor analysis. P < 0.05 indicated the significant difference.

## Results

Finally, 148 patients with surgically treated lung tumors were included in the study according to the inclusion and exclusion criteria. The average HAD score of the 148 patients was (12.31 ± 3.56), including 45 cases without anxiety and depression, and 103 cases with anxiety and depression, accounting for 30.41 and 69.59%, respectively.

As shown in Table 1, age, gender, and marital status had no correlation with the perioperative psychological status of patients with lung cancer (*P* > 0.05). Income status, medical environment, worried about work, psychological state of family members, family care level, sleep quality, history of mental illness, worried about postoperative pain, patients' knowledge of the diagnosis of the condition and nature of tumor were the single factors that affected the psychological state of patients with lung tumor during perioperative period (*P* < 0.05).

**Table 1:** Univariate analysis of psychological status of patients with pulmonary tumor during perioperative period.

**Clinical pathological features**		**With anxiety and depression group (*n* = 103)**	**Without anxiety and depression group (*n* = 45)**	***t/χ^2^* value**	***P-*value**
Age		49.31 ± 9.34	49.87 ± 9.71	0.332	0.741
Gender	Male	61(59.22)	25(55.56)	0.173	0.677
	Female	42(40.78)	20(44.44)		
Marital status	Married	69(66.99)	24(53.33)	5.287	0.071
	Unmarried	23(22.33)	13(28.89)		
	Widowed	11(10.68)	8(17.78)		
Income status	>10,000 yuan/month	29(28.16)	23(51.11)	9.868	0.007
	5,000~10,000 yuan/month	19(18.45)	10(22.22)		
	<5,000 yuan/month	55(53.40)	12(26.67)		
Medical environment	Good	40(38.83)	35(77.78)	19.001	<0.001
	Poor	63(61.17)	10(22.22)		
Worried about work	No	32(31.07)	30(66.67)	16.304	<0.001
	Yes	71(68.93)	15(33.33)		
Psychological state of family members	Without anxiety and depression	21(20.39)	34(75.56)	41.341	<0.001
	Anxiety and depression	83(80.58)	11(24.44)		
Family concern	Good	34(33.01)	41(91.11)	42.296	<0.001
	Poor	69(66.99)	4(8.89)		
Sleep quality	Good	32(31.07)	32(71.11)	20.460	<0.001
	Poor	71(68.93)	13(28.89)		
History of mental illness	No	66(64.08)	42(93.33)	13.591	<0.001
	Yes	37(35.92)	3(6.67)		
Worried about postoperative pain	No	20(19.42)	32(71.11)	36.721	<0.001
	Yes	83(80.58)	13(28.89)		
Patient's knowledge of their condition	Clear	47(45.63)	37(82.22)	17.085	<0.001
	Fuzziness	56(54.37)	8(17.78)		
Tumor nature	Benign	43(41.75)	29(64.44)	6.458	0.011
	Malignant	60(58.25)	16(35.56)		

The depression and anxiety status of patients were taken as dependent variables, and the factors with significant differences in Table 1 were taken as independent variables to be included in the Logistic regression model. The assignments of the dependent variable and independent variable are shown in Table 2.

**Table 2:** Variable assignment table of multi-factor analysis of depression and anxiety of patients with pulmonary tumor during perioperative period.

**Variable**	**The assignment**
**Dependent variable**
Anxiety and depression	No = 0, Yes = 1
**Independent variables**
Income status	>10,000 yuan/month = 0, 5,000~10,000 yuan/month = 1, <5,000 yuan/month = 2
Medical environment	Good = 0, Poor = 1
Worried about work	No = 0, Yes = 1
Psychological state of family members	Without anxiety and depression = 0, With anxiety and depression = 1
Family concern	No = 0, Yes = 1
Sleep quality	Good = 0, Poor = 1
History of mental illness	No = 0, Yes = 1
Worried about postoperative pain	No = 0, Yes = 1
Patient's knowledge of their condition	Clear = 0, Fuzziness = 1
Tumor nature	Benign = 0, Malignant = 1

As shown in Table 3, income status, psychological status of family members, sleep quality, worried about postoperative pain and nature of tumor were the independent risk factors for psychological status of patients with pulmonary tumor during perioperative period (*P* < 0.05).

**Table 3:** Multivariate logistic regression analysis of of psychological status of patients with pulmonary tumor during perioperative period.

**Factors**	**β**	**SE**	**Wald**	***P***	**OR(95%CI)**
Income status	0.269	0.036	36.026	0.006	1.285~10.349
Medical environment	0.264	0.989	37.169	0.461	0.615~2.461
Worried about work	0.349	0.761	42.590	0.339	0.479~1.582
Psychological state of family members	0.152	0.027	39.462	0.043	1.933~11.597
Family concern	0.139	0.633	30.481	0.085	0.346~1.274
Sleep quality	0.136	0.031	31.298	0.013	1.088~9.315
History of mental illness	0.264	0.648	42.091	0.951	0.218~1.979
Worried about postoperative pain	0.265	0.041	46.440	<0.001	1.798~7.511
Patient's knowledge of their condition	0.281	0.711	32.899	1.563	0.634~3.491
Tumor nature	0.165	0.036	20.592	<0.001	1.591~6.949
Constant	−2.649	0.165	32.792	0.000	-

Of the 148 patients, 74 received traditional care (control group) and 74 received narrative care in combination with SFA (study group). In the control group, there were 23 cases without anxiety and depression and 51 cases with anxiety and depression. There were 22 cases without anxiety and depression and 52 cases with anxiety and depression in the study group. There was no significant difference in anxiety and depression between the two groups (*P* > 0.05).

As shown in Table 4, there was no difference in HAD score, medication behavior, INR monitoring behavior and life behavior score between the two groups at the time of admission (*P* > 0.05). At the time of discharge, the HAD scores of the two groups were decreased, and those in the study group were lower than those in the control group (*P* < 0.05). At the time of discharge, medication behavior, INR monitoring behavior and life behavior of the two groups were increased, and the study group was higher than the control group (*P* < 0.05).

**Table 4:** Comparison of HAD scores and Compliance Behavior scores of patients with different nursing schemes before and after nursing.

**Group**	***n***	**HAD**		**Medication behavior**		**INR monitoring behavior**		**Life behavior**		**Aggregate score**	
		**Before nursing**	**After nursing**	**Before nursing**	**After nursing**	**Before nursing**	**After nursing**	**Before nursing**	**After nursing**	**Before nursing**	**After nursing**
Study group	74	12.96 ± 3.34	7.05 ± 2.16	6.39 ± 0.36	7.87 ± 0.65	6.41 ± 1.21	7.94 ± 1.35	8.69 ± 1.25	10.69 ± 1.63	21.49 ± 2.82	26.50 ± 3.63
control group	74	12.61 ± 3.45	8.94 ± 2.31	6.25 ± 0.41	7.13 ± 0.34	6.53 ± 1.25	7.42 ± 1.12	8.81 ± 1.32	9.67 ± 1.34	21.59 ± 2.98	24.22 ± 2.80
T		0.627	5.141	0.631	8.678	0.593	2.599	0.568	4.158	0.210	4.278
P		0.532	<0.001	0.529	<0.001	0.554	0.010	0.571	<0.001	0.834	<0.001

## Discussion

Anxiety, depression, fear and other abnormal emotions often occur to patients with lung tumors when they recognize their condition and the lung lobe needs to be removed during surgery (8). Studies have shown that between 25 and 70% of hospitalized patients have both anxiety and depression (9). In this study, HAD was used to assess the psychological state of patients with lung cancer during the perioperative period. The results showed that the proportion of anxiety and depression was 69.59%, which was basically in line with the previous literature (10).

### Analysis of Factors Affecting Perioperative Patients' Psychological State

The logistics regression analysis was used to study the correlation between each independent variable and the occurrence of anxiety and depression in patients. Among them, income status, sleep quality, psychological status of family members, worried about postoperative pain and nature of tumor were the independent risk factors for psychological status of patients with pulmonary tumor during perioperative period (*P* < 0.05). There are many surgical options for lung tumors, and minimally invasive diagnostic techniques are being continually improved. Although various schemes have shown certain efficacy, the operation and treatment costs still impose certain pressure on patients. Especially for patients with a large gap between economic income and medical expenses, they may even be poor due to illness (11). This phenomenon also aggravates people's psychological endurance day by day, thus inducing patients' emotions to be in a negative state (12). Therefore, it is necessary to formulate treatment strategies according to the economic conditions of patients in clinical treatment, and it can help patients with low economic ability to seek social help, to reduce the impact of economic income on the psychological state of patients as much as possible.

A large number of studies have confirmed the correlation between sleep quality and mental health status (13). On the one hand, poor quality of time and sleep will lead to decreased body immunity and affect the curative effect of surgery (14). On the other hand, people with poor sleep quality tend to look at things in a negative attitude, which leads to dissatisfaction, disgust, depression and other negative emotional reactions. Therefore, sleep propaganda and education for patients should be strengthened in nursing work, and sedative drugs should be applied when necessary.

Acute trauma and internal organ damage caused by the operation itself and stimulation of drainage can lead to postoperative pain, which can cause severe physiological and psychological reactions and lead to a decline in the quality of life of the patient (15). Patients often have negative psychology for fear of postoperative pain, so targeted control of symptoms and maintaining comfort are the main nursing countermeasures (16).

Patients with lung tumors should not only face the pressure brought by tumors and surgery, but also face the psychological burden brought by a series of negative life events (17). In addition, the family members of patients with lung cancer should also be concerned about the impact. The anxiety and depression degree of family members will not only affect their physical and mental health, but also have a great negative effect on the patient's psychology, treatment and even future rehabilitation. Therefore, the psychological reactions of family members cannot be ignored in the process of clinical diagnosis and treatment. At the same time of nursing patients, psychological intervention should be carried out for their families.

Compared with benign tumors, patients with malignant tumors are under double mental pressure of cancer diagnosis and treatment during the treatment stage. Surgical resection of a wide range often affects the normal function of the body or the organ in which the tumor is located. Based on this, nursing staff should deeply understand the psychological changes of patients and assist doctors to patiently explain the necessity of the operation to save lives and prevent tumor recurrence before operation (18). In addition, patients with malignant tumors often need to receive adjuvant treatments such as radiotherapy and chemotherapy after operation, and their anxiety is often aggravated by their adjuvant treatments. Therefore, before carrying out various treatments, explanation should be carefully done so that patients can understand the role of treatment, brief steps, possible side effects and matters needing cooperation are links that cannot be ignored in psychological care for malignant tumors.

### Narrative Nursing Combined With SFA Can Effectively Improve Anxiety and Depression in Patients With Pulmonary Tumor During Perioperative Period

The traditional perioperative care focuses on health education to patients and active prevention of postoperative complications, ignoring the stimulation and cultivation of patients' potential to participate in disease treatment and nursing. SFA is a clinical intervention model that fully respects individuals, and its characteristics are to guide individuals to fully mobilize their resources and potential, and take the initiative to participate in behavioral changes (19). The key of the SFA is to assist patients to construct constructive solutions, utilize their own resources and potential, and improve the puzzles and problems encountered in the current care (20). Some scholars believe that the implementation of the SFA is based on two aspects. One is to establish a specific and feasible goal based on the individual thinking mode, and the other is to use the exceptional question to explore the individual's experience in dealing with the problem and to seek various resources to achieve the goal. Narrative nursing therapy is a kind of treatment in postmodern psychology. At present, the specific definition of narrative nursing is still vague. Based on the views of many literatures, we have summarized the concept of narrative nursing as a nursing practice method in which nursing staff listen to the stories of patients, discover the key points of nursing and then implement nursing intervention on patients to help patients achieve the purpose of reconstructing life and diseases. Narrative nursing can externalize patients' bad emotions through effective communication and deconstruct their inner anxiety problems in empathy with patients on the basis of establishing good nurse-patient relationship (21–23). In this study, at the time of discharge, the HAD score of the study group was lower than that of the control group, and the medication behavior, INR monitoring behavior and life behavior were higher than those of the control group (*P* < 0.05). It indicated that narrative nursing combined with SFA could effectively improve the anxiety and depression status of patients with lung cancer during the perioperative period and enhance the treatment compliance of patients.

## Conclusion

In summary, income status, sleep quality, fear of postoperative pain, Patient's knowledge of their condition and nature of tumor are the independent risk factors affecting the psychological state of patients with lung tumor during perioperative period. Narrative nursing combined with focused solution model can effectively improve the anxiety and depression status of patients with lung tumor during perioperative period and enhance their compliance behavior, which is worthy of promotion.

## Data Availability Statement

The original contributions presented in the study are included in the article/supplementary material, further inquiries can be directed to the corresponding author/s.

## Ethics Statement

The studies involving human participants were reviewed and approved by the Ethics Committee of the Shanghai Chest Hospital. The patients/participants provided their written informed consent to participate in this study.

## Author Contributions

LG is responsible for the design of the article. FJ is responsible for the evaluation and testing of the results. JF is responsible for data statistics and the writing of the paper. LJ is the supervisor of the entire study. All authors contributed to the article and approved the submitted version.

## Funding

This research was supported by the backbone of scientific research in Shanghai Nursing Plateau Discipline Construction Hundred Talents Program (Hlgy1828kygg).

## Conflict of Interest

The authors declare that the research was conducted in the absence of any commercial or financial relationships that could be construed as a potential conflict of interest.

## Publisher's Note

All claims expressed in this article are solely those of the authors and do not necessarily represent those of their affiliated organizations, or those of the publisher, the editors and the reviewers. Any product that may be evaluated in this article, or claim that may be made by its manufacturer, is not guaranteed or endorsed by the publisher.
